# The Power Laws of Violence against Women: Rescaling Research and Policies

**DOI:** 10.1371/journal.pone.0040289

**Published:** 2012-07-02

**Authors:** Karolin E. Kappler, Andreas Kaltenbrunner

**Affiliations:** Barcelona Media – Innovation Centre, Barcelona, Spain; Tulane University, United States of America

## Abstract

**Background:**

Violence against Women –despite its perpetuation over centuries and its omnipresence at all social levels– entered into social consciousness and the general agenda of Social Sciences only recently, mainly thanks to feminist research, campaigns, and general social awareness. The present article analyzes in a secondary analysis of German prevalence data on Violence against Women, whether the frequency and severity of Violence against Women can be described with power laws.

**Principal Findings:**

Although the investigated distributions all resemble power-law distributions, a rigorous statistical analysis accepts this hypothesis at a significance level of 0.1 only for 1 of 5 cases of the tested frequency distributions and with some restrictions for the severity of physical violence. Lowering the significance level to 0.01 leads to the acceptance of the power-law hypothesis in 2 of the 5 tested frequency distributions and as well for the severity of domestic violence. The rejections might be mainly due to the noise in the data, with biases caused by self-reporting, errors through rounding, desirability response bias, and selection bias.

**Conclusion:**

Future victimological surveys should be designed explicitly to avoid these deficiencies in the data to be able to clearly answer the question whether Violence against Women follows a power-law pattern. This finding would not only have statistical implications for the processing and presentation of the data, but also groundbreaking consequences on the general understanding of Violence against Women and policy modeling, as the skewed nature of the underlying distributions makes evident that Violence against Women is a highly disparate and unequal social problem. This opens new questions for interdisciplinary research, regarding the interplay between environmental, experimental, and social factors on victimization.

## Introduction

Capturing a clear picture of Violence against Women (VaW), both in numbers and in severity, has proved a major challenge in Social Sciences, because of the difficulties in accessing the affected population and the data generally being based on retrospective accounts. Numerous national prevalence studies [1] –mainly carried out in the 90s and early 2000s– have partly solved this challenge, quantifying VaW and showing its social impact. Curiously and despite this knowledge, the statistical distribution of VaW has never been taken into consideration, although it might provide a deeper insight into a major social problem which is still unsolved. This fact is even more surprising as power-law distributions are omnipresent in many human activities [2], inter alia in other violent social phenomena, such as the number of casualties in wars [3], the severity of terror-attacks [4],[5] or human insurgency [6]. This confirms the fact that research on VaW is treated and published separately from other studies on violence, slowing down the progress of scientific research and social knowledge, as inter- and transdisciplinary synergies are rarely exploited.

To partly overcome this gap between different disciplines, we investigate, in a secondary analysis with data from the German prevalence study on VaW [7], whether the distribution of the number of times a women is victim of a certain type of violence and its severity scale can be described by a discrete power-law distribution.

## Materials and Methods

### Description of Data Set

**Figure 1:**
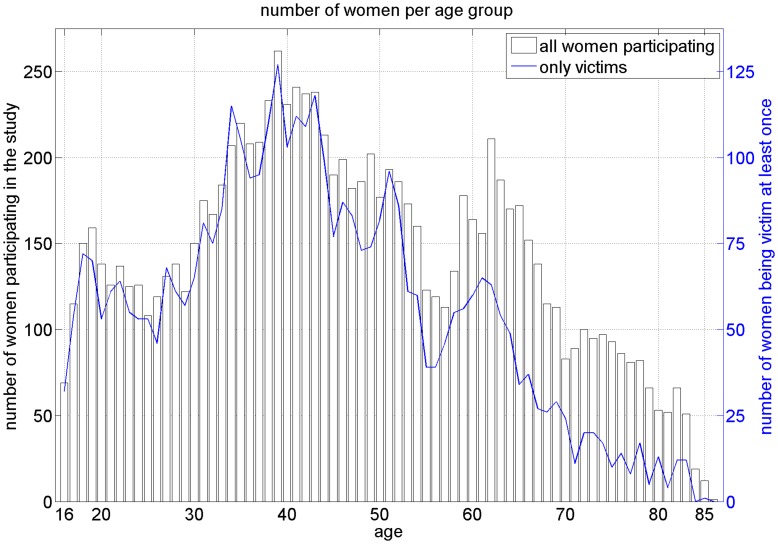
Age distribution of the women pariticipating in the study. Black bars indicate the number of women per age group participating in the study. The blue line refers to the age distribution of the women, who have reported experiencing at least one violent strike (restricted to the data analyzed in the current study).

We carry out a secondary analysis with data from the research study “Health, Well-Being and Personal Safety of Women in Germany” [7]. It was the first representative survey on VaW in Germany, forming part of the national action plan published in 1999 by the German Federal Government to combat VaW. The representative study is based on 10,264 interviews, conducted nation-wide from February until October 2003 with women aged 16 to 85, residing in Germany. The data was drawn from a basic representative sample; the rate of yield in the gross random sample adjusted for neutral omission amounted to 52%. The average age of the interviewees is 46.7 years and the age distribution of the whole sample, including all women and the sub-sample of women, experiencing violence at least once, is shown in Figure 1. The sub-sample includes all women, who declared to be a victim in at least one of the questions studied in the current article.

The women were interviewed extensively as to their experiences with violence, their feelings of personal safety and their psychosocial and physical health situations. Following other international prevalence studies [8], [9], the data were collected in a two-step approach, to better illuminate grey areas related to sensitive and problematic types of violence and abuse, such as sexual violence or violence occurring in family contexts and partner relationship.

First, a standardized, 60–90 minutes face-to-face interviews took place either in the interviewees’ homes or, in some cases, at other locations. Second, these oral interviews were supplemented by self-administered written questionnaires on family and partner violence, completed by the interviewees themselves, after finishing the oral interviews.

Central forms of violence included in this study are physical violence (including any kind of physical aggression, such as slapping in the face, hair pulling, battering, kicking or threatening with a weapon), sexual violence (according to the German penal code including any forced sexual act, such as rape or sexual assault, carried out against the victim’s will through threats or physical force), sexual harassment (including any type of intimidation, bullying or coercion of a sexual nature, as well as the unwelcome or inappropriate promise of rewards in exchange for sexual favor), and psychological abuse (including any kind of offensive or distressing experience, such as insults, pejorative comments, or humiliations). Women were first asked about their experience of each of these four forms of violence since the age of 16 by way of a generalized question in the oral interview. This was followed, if any abuse record was confirmed, by further questions on frequency and impact of the violence, victim-abuser relationship, and further details about the concrete situations in which it occurred.

Physical, sexual and psychological abuse were then addressed in the written questionnaire, related both to violence by current or former male and female partners, as well as abuse experienced in childhood and adolescence up to the age of 16.

**Figure 2:**
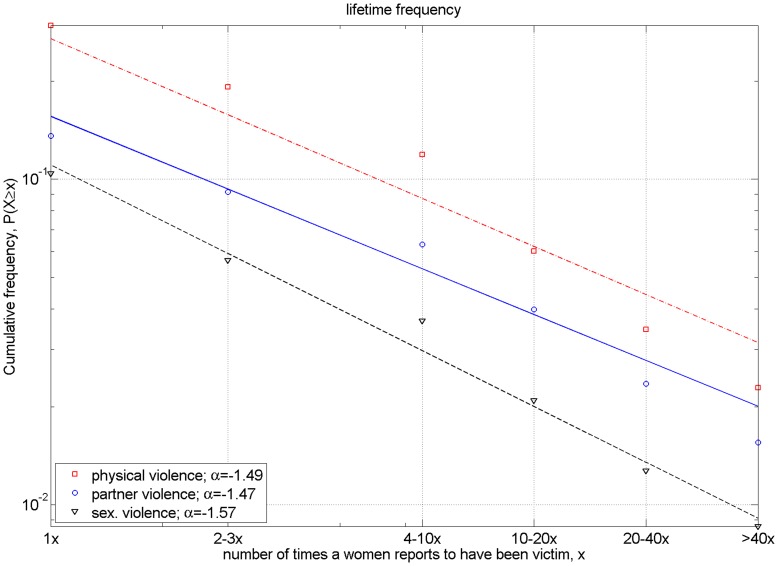
Complementary cumulative distribution of the number of reports per women of different forms of violence experienced since the age of 16. ccdf of the number of reports per woman of physical violence (red squares), partner violence (blue circles), and sexual violence (black triangles) since the age of 16. Adjusted power laws (lines in the corresponding color) have exponents 1.49, 1.47 and 1.57 respectively. The latest data point (<40x) has been placed at x = 80 to continue the logarithmic binning of the previous 2 bins. This has been done purely for illustration and has had no influence in the reported power-law fits nor in the statistical test, as this data bin has been omitted there.

**Figure 3:**
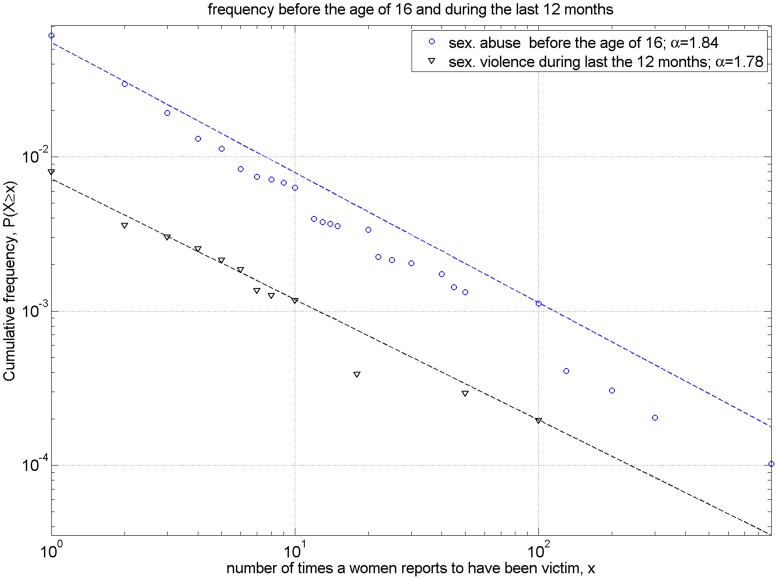
Complementary cumulative distribution of the number of reports per women of different forms of violence. Complementary cumulative distribution (ccdf) of the number of reports per women of sexual abuse occurring before their 16^th^ birthday (blue circles) or sexual violence occurring during the last 12 months (black triangles). Adjusted power laws (dashed lines in the corresponding color) have exponents 1.84 and 1.78.

### Access to Data Set

The data of the prevalence study are stored at the Data Archive for the Social Sciences – Gesis Leibniz Institute for Social Sciences (the former Zentralarchiv für Empirische Sozialforschung ZA). This data archive provides data service for national and international comparative surveys, making data sets accessible to interested researchers. For further information on and direct access to the data set used in this study, one can contact directly the data archive services: http://www.gesis.org/en/services/data-analysis/data-archive-service/The data set is stored and available under the following codes: ZA4193 for the main study and ZA4194 for the supplementary study. (In German: Lebenssituation, Sicherheit und Gesundheit von Frauen in Deutschland. Gewalt gegen Frauen – Haupt- und Zusatzbefragung.).

### Description of Data Used in Secondary Analysis

We limit our analysis on the frequency and severity of violence (including both the face-to-face interview and the written questionnaire). Other data provided by the study, such as the frequencies of other types of violence, e.g. sexual harassment at work, or the severity-scales of sexual violence, had to be excluded, because it offers too generic, fragmented, or scarce information, lacking precise quantitative data or including only a few items in the severity-list.

**Figure 4:**
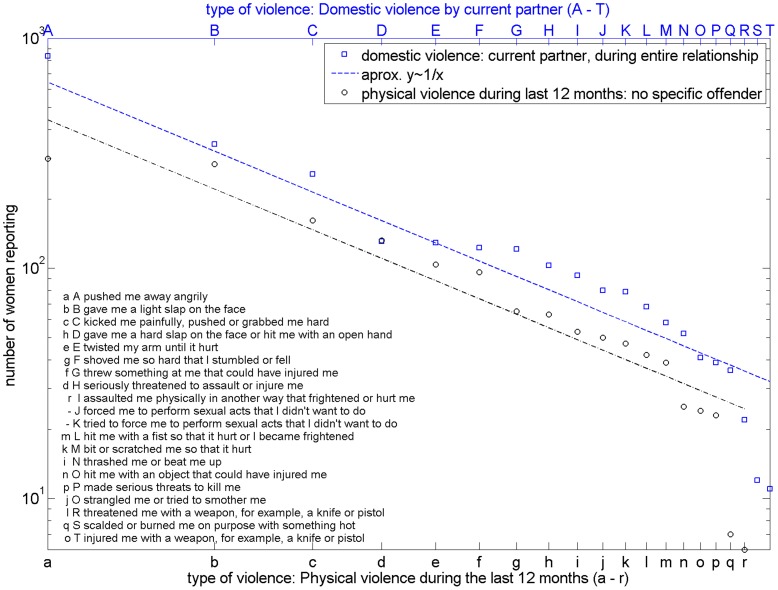
Distributions of the severity of violence suffered by women aligned by their frequency. Distributions of the severity of violence suffered by women aligned by their frequency of accounts in the context of (a) domestic violence by current partner (blue squares) or (b) physical violence during the last 12 months (black circles). Apart from the less frequent items, a good coincidence between frequency of accounts and power laws with exponent 1 (dashed lines) can be observed. Uppercase letters encode the items of domestic violence by current partner (during the entire duration of the relationship) and lowercase letters the items of physical violence (no specific offender) during the last 12 months.

Three different types of questions regarding the **frequency of violent experience** can be found in the dataset.

- The first group does not ask for precise numbers but for adverbs, such as ‘often’, ‘occasionally’, ‘rarely’, ‘once’ and ‘never’. We did not analyze these questions due to its lack of numeric precision.
- The second group includes binned answers about the total number of times the women have been victims of a certain type of violence in their entire lifetime. In this case, possible answers only include the following 7 frequency ranges: never, once, 2–3 times, 4–10 times, 10–20 times, 20–40 times and more than 40 times. Note that the boundaries of the ranges are overlapping. E.g. a person being victim 10 times could correspond either in the 4–10 or the 10–20 bin. This seems to be a deficiency in the design of the questionnaire. For our analysis, we assume the following bins: 0, 1, 2–3, 4–10, 11–20, 21–40, >40.
- The third group of questions asks for the interviewees’ precise estimate of number of violent events suffered.

**Table 1:** Size of the data-set.

Question	Total number of women who answered the question	Number of women who have been a victim at least once	Percentage
sex. Abuse before 16	9825	602	6.13%
sex. Violence during the last 12 months	10264	82	0.80%
physical violence	10178	3022	29.69%
partner violence	10015	1360	13.58%
sex. violence	10236	1065	10.40%
severity: domestic violence	9640	859	8.91%
severity: physical violence	10264	546	5.32%

Columns indicate: Type of violence or severity question, the number of women answering the question, and the number of women who report at least one incidence of violence in the corresponding question. The last column gives the percentage of victims with respect to the values of the second column.

In Figure 2, we depict three questions of the second group with the binned data (questions n° 705, n° 16, and n° 807 of the original questionnaire) and in Figure 3, we show the data of two exemplary questions of the third group with open answers (questions n° 73 and 805 of the original questionnaire). Furthermore, Figure 2 refers to violent events suffered during the women’s lifetime period (since age 16), while Figure 3 shows the number of sexual abuses suffered until age 16 and of sexual violence experienced during the last year.

**Table 2:** Cross table of the overlap between the victims of the different types of violence.

	sex. Abusebefore 16	sex. Violence duringthe last 12 months	physical violence	partner violence	sex. violence	severity: domesticviolence	severity: physicalviolence
sex. Abuse before 16	–	8	330	217	205	117	70
sex. Violence during the last 12 months	8	–	64	30	81	20	44
physical violence	330	64	–	1091	736	552	518
partner violence	217	30	1091	–	502	353	193
sex. violence	205	81	736	502	–	170	141
severity: domestic violence	117	20	552	353	170	–	162
severity: physical violence	70	44	518	193	141	162	–

The values indicate the number of women who report having at least one incidence in both of the two corresponding questions.

A fourth group of questions deals with the **severity of violent experiences**, i.e. the specific item of violence, as women are victimized by kicking, slapping, pushing, forced sexual acts, etc. The possible answers are ‘never’, ‘once’ or ‘several times’. We merged the ‘once’ and ‘several times’ answers, obtaining dichotomist ‘yes’/‘no’-data, whether the women have experienced a specific violence-item in the context of domestic violence or physical violence. The rank-size distributions of the number of women who have experienced a certain item are then the subject of analysis.

The questions present the following items’ lists to the interviewees:

*List of items* (Figure 4) for domestic violence by current partners during entire relationship (A – T; question n° 12 of original questionnaire) and physical violence during the last 12 months (no specific offender) (a – r; question n° 701 of original questionnaire):

‘A/a- pushed me away angrily’

‘B/b- gave me a light slap on the face’

‘C/c- kicked me painfully, pushed or grabbed me hard’

‘D/h- gave me a hard slap on the face or hit me with an open hand’

‘E/e- twisted my arm until it hurt’

‘F/g- shoved me so hard that I stumbled or fell’

‘G/f- threw something at me that could have injured me’

‘H/d- seriously threatened to assault or injure me’

‘I/r- assaulted me physically in another way that frightened or hurt me’

‘J/− - forced me to perform sexual acts that I didn’t want to do’

‘K/− - tried to force me to perform sexual acts that I didn’t want to do’

‘L/m- hit me with a fist so that it hurt or I became frightened’

‘M/k- bit or scratched me so that it hurt’

‘N/i- thrashed me or beat me up’

‘O/n- hit me with an object that could have injured me’

‘P/p- made serious threats to kill me’

‘Q/j- strangled me or tried to smother me’

‘R/l- threatened me with a weapon, for example, a knife or pistol’

‘S/q- scalded or burned me on purpose with something hot’

‘T/o- injured me with a weapon, for example, a knife or pistol’

**Table 3:** Results of parameters estimation and the statistical tests of the PL-fits.

Dataset	*α*	KS-test	PL test of Clauset et al. [2]
Physical violence (Figure 2)	1.490 (estimated with max. likelihood)	p<10^−5^	p = 0.000 reject
Partner violence (Figure 2)	1.470 (estimated with max. likelihood)	p<10^−4^	p = 0.000 reject
Sex. violence (Figure 2)	1.570 (estimated with max. likelihood)	p = 0.13 for *α* estimated	p = 0.008 reject
Sexual Abuse before the ageof 16 (Figure 3)	1.844 (estimated with max. likelihood)	p = 0.48 for *α* estimated	p = 0.04 reject at significance level 0.1, accept at significance level 0.01
Sexual violence during the last12 months (Figure 3)	1.782 (estimated with max. likelihood)	p = 0.65 for *α* estimated	p = 013 accept at significance level 0.1
Domestic violence (Figure 4)	1.019 (calculated with least-squares fitting)	p = 0.03 for *α = 1, x_min_ = 2* andremoving the last 3 data points,reject at significance level 0.1,accept at significance level 0.01	Not applicable
Physical violence (Figure 4)	0.996 (calculated with least-squares fitting)	p = 0.13 for *α = 1, x_min_ = 2* andremoving the last 2 data points,accept at significance level 0.1	Not applicable

The results of the KS test are only valid in the case of the data presented in Figure 4 (bottom two rows). The test procedure provided by Clauset et al [2] is not applicable on rank frequency distributions (nor on power laws with exponent 1). We fixate *x_min_ = 1* if not stated otherwise.

### Data Aggregation and Overlap

Not all women participating answer all questions. The exact size of the dataset, i.e. the number of women who answer the questions described above is given in Table 1. The table also gives the number and corresponding percentage of women who report having been victim at least once of a specific type of violence. Depending on the question this percentage varies between 30% of women who have experienced at least once in their lifetime physical violence and 0.8% who have been victim of sexual violence in the 12 months before the study.

The number of women who report being victim in two different specific questions is given in Table 2. The types of violence asked for in the different questions are not exclusive nor is one a clear subset of another; e.g. cases of partner violence may also reported under sexual violence or physical violence and vice versa. Consequently, any kind of data aggregation is not possible with the current data.

### Methodological Approach

We investigate whether the distribution of the number of times a woman is victim of a certain type of violence can be described by a discrete power-law distribution. That is, whether the probability of being a victim *x* times is proportional to *x^−α^*, or more exactly whether *P(X = x) = x^−α^/ζ(α,x_min_)* where *ζ(α,x_min_)* is the generalized zeta function and *x_min_ >0* the lower bound of the power-law behavior. The complementary cumulative distribution function or CCDF is in this case *P(X≥x) = ζ(α,x)_/_ζ(α,x_min_).* The CCDF of empirical data allows to reduce the usually large fluctuations of the less frequent items in the tail of a power-law distribution. If we furthermore approximate the discrete power-law distribution with its continuous counterpart we can use that *P(X≥x)∼ x^−α+1^*, i.e. a power law with an exponent which is smaller by 1 than the exponent of the underlying distribution.

### Parameter Estimation and Statistical test

For the datasets of group 2 and 3, we use maximum likelihood estimations as proposed by Clauset et al. in [2] to extract the exponent *α* of a power-law fit of the data. To test the hypothesis that the corresponding distributions follow power laws we use the discrete power-law test proposed in [2]. We fixate *x_min_ = 1* for this two datasets.

For the datasets of group 4, we test the hypothesis that the underlying data can be described by a power law with exponent *α = 1* with a Kolmogorv-Smirnoff (KS) test. Note that the result of a KS-test is invalid when the fitted distribution uses parameters estimated from the data, however we do not perform a parameter extraction in the case of the datasets of group 4.

The results (p-values) of the two statistical tests are given in Table 3.

### Limitations of the Data

The data set is quite noisy due to its survey provenance. As discussed in the vast literature on survey methods on health, sexual and marketing topics [10]–[13], there exist specific biases related to self-reporting of negative and undesirable events, such as errors based on rounding effects, underreporting, selection and recall processes. Victims often avoid recalling the negative and unpleasant events or they are not able to remember any of the violence they experienced, as they suffer a complete memory-loss. Although there is no proof of the existence of such biases in the used data, its influence can be assumed and could partly explain the noisiness of the data and the deviations of the ideal power-law distributions, which coincide with rounded numbers, such as 10 or 100. In the case of the 12 months prevalence, the distribution drops towards the right, as the frequency of incidents is limited through the restricted time lapse of 365 days. Nevertheless and until better data is available, the prevalence studies give still the best insight into a little known phenomenon.

## Results

Our results indicate that, although both the frequencies and the severity of VaW resemble power-law distributions, rigorous statistical testing accepts this hypothesis with a significance level of 0.1 only for the case of the victims of sexual violence during the last 12 months and with some restrictions for the severity of physical violence. More exactly, maximum likelihood estimation fits a power law with exponent of approximately 1.5 on the distribution of the number of times women are victims of physical, partner or sexual violence during their lifetime. Figure 2 depicts in logarithmic scale the corresponding complementary cumulative distributions which show the proportion of women who report having suffered at least x-times the corresponding form of violence. The larger than expected values (if an underlying power-law distribution is assumed) of the reported frequencies in the 4–10 times bin are the main cause that the power-law fits are rejected as a valid model for the underlying distribution.

The power-law approximations of the distributions of the number of times women suffer sexual abuse in childhood before the age of 16 and sexual violence during the last 12 months (depicted in Figure 3) are steeper power laws with exponents around 1.8. In this case, the power-law hypothesis is accepted for the distribution of sexual violence during the last 12 months with significance level 0.1, but rejected for the distribution of sexual abuse (the corresponding p-values are given in Table 3). Lowering the significance level to 0.01 would accept the power-law hypothesis as well in the later case. The highly skewed distributions show that VaW represents a diverse and unequal social phenomenon, characterized by the inexistence of a typical and average case and the frequent existence of outliers, which carry most events, while most are below average.

The steeper power-law coefficient for sexual violence implies that it is less likely to observe women reporting large frequencies than for the two other types of violence. This might be caused by the less common and more stigmatized character of sexual violence.

Another type of power-law like distribution (Fig. 4, with exponent 1) is found in the frequency-rank distributions of the different items of domestic violence by current partners and general physical violence. A KS-test confirms the power-law fit for physical violence at a 0.1 significance level and for domestic violence at the 0.01 level, if the most frequent item (''pushed me away angrily”) and the items with very low frequencies are omitted.

It is interesting to notice that the order of the items seems to coincide with a hypothetical ordering by severity. The less severe items are more frequent and the following items are mediated by the experience of pain, fear and fear of death and the use of objects and weapons, such as represented by the answers n, o, p, q and r for experienced events of physical violence during the last 12 months (no specific offender) and N, O, P, Q, R, S, and T for domestic violence suffered by current partner during entire relationship. The distribution drops towards the left of this data. This might be explained through a specific cultural bias, in which violent acts, such as burns or the use of weapons are less frequent, as they are either known from other cultural contexts as in the first case or because of the specific German firearms law as in the second case.

A power law with exponent 1 is often found for word rank-frequency distributions and can be explained by optimizing the amount of information transmitted in a given language [14]. Recent research findings on VaW [15] describe the perpetration of gender violence as an act of normative power exerted by the aggressor on the victim through (re)naming and (re)defining the violent event in his own terms, negating the victim any communicative power and the use of her own words. The exponent 1 fits to this recent qualitative finding and supports the claim to understand VaW –at least partly– as a communicative event, which depends mainly on a power struggle of defining the act as violent or not.

## Discussion

This is the first time that a study investigates whether VaW follows a power law. We find evidence that at least the frequency distribution of experiencing sexual violence during the last 12 months follows a power law. Rejections of the power-law hypothesis for other frequency distributions may be caused by the large amount of noise and effects caused by the self-reported data, like underreporting due to the victims’ memory-loss, rounding-effects, selection or recall processes of negative and undesirable events. Future studies, which manage to overcome this limitation in the data, might well find a better coincidence for the described phenomenon with power laws or related distributions. Consequently, the way data is collected should help to reduce the noise. Apart from avoiding overlapping time intervals, the impossibility to aggregate the data is a further inconvenient, which should be ameliorated in future research.

Furthermore, although a power-law fit cannot be confirmed in most cases at the significance level of 0.1 recommend by Clauset et al. in [2], VaW seems to be a highly skewed, heavy tailed phenomenon, quantifying the increased risk of extreme victimizations, while these high-risk areas are generally omitted if a Gaussian-distribution is assumed as is generally done in Social Sciences. Note especially that all the exponents of the power-law fits reported here are lower than 2, which implies that the mean and standard deviation do not exist in a strict mathematical sense, as they are infinite [16]. If the power law (or related distribution) nature of VaW were confirmed by future studies, this would imply that any means and standard deviations calculated from these surveys would not have any descriptive value. Consequently, as we raise this suspicion with our study, they should not be used to compare different studies or even different instances of the same study. Hence, our findings have a major impact on the handling of the prevalence data on VaW. The discussions on standards and good practices in data collection on VaW [17] and the permanently carried out prevalence studies all around the world show the crucial importance of this new insight.

Considering VaW from a social and systemic perspective, a power-law resemblance –especially with exponent 1– would point to the specific communicative value of VaW for the victims and for society. Paradoxically, power laws often emerge in contexts where diversity, freedom of choice and preferences generate inequality and diversity, while violence is generally considered a morally and legally condemnable event, not getting associated with any act of preference and positive choice. A possible hypothesis to explain this paradox is a specific communicative link between power and violence: In a society, which is characterized by power and power play, power is associated with a rather positive value. And as seen previously and from a constructivist point of view, violence can be understood as a communicative act of power. Hence, the existence of power-law distributions in VaW-data would become understandable. The “surprising link between violent and non-violent forms of human behaviour” as discussed in [6] could be partly explained through such findings. This might provide a further argument to describe the still mysterious interplay between social and hereditary factors which seem to influence women’s proneness to VaW [18] and offer a new element in the vivid research on resilience factors [19].

Moreover, the strong heterogeneity of the victimization data completely opens up new insights for policy modelling, because a single and unique prevention or intervention strategy cannot cover the large variety in the different dimensions of victimizations, ignoring the heavy tails of the distributions. Thus, it should be reconsidered under this new perspective and reoriented towards specific cohorts or niches on the frecuency scale, an insight which is of particular importance in times of social cuts and policy reorganization. The calculation of recognized measures of diversity and statistical dispersion, such as the Gini coefficient [20], might offer new aspects for the analysis of resilience factors [17]. All these possible insights may help policy makers to better assess the extent and distribution of VaW, distinguishing the risk of different population groups [21], in order to set up specific and more adequate prevention and intervention programs.

In conclusion, the heavy tail of VaW challenges both researchers and policy makers and opens up new perspectives for interdisciplinary cooperation.
